# Effect of monovalent COVID-19 vaccines on viral interference between SARS-CoV-2 and several DNA viruses in patients with long-COVID syndrome

**DOI:** 10.1038/s41541-023-00739-2

**Published:** 2023-09-29

**Authors:** Mariann Gyöngyösi, Dominika Lukovic, Julia Mester-Tonczar, Katrin Zlabinger, Patrick Einzinger, Andreas Spannbauer, Victor Schweiger, Katharina Schefberger, Eslam Samaha, Jutta Bergler-Klein, Martin Riesenhuber, Christian Nitsche, Christian Hengstenberg, Patrick Mucher, Helmuth Haslacher, Monika Breuer, Robert Strassl, Elisabeth Puchhammer-Stöckl, Christian Loewe, Dietrich Beitzke, Ena Hasimbegovic, Thomas A. Zelniker

**Affiliations:** 1 Division of Cardiology, Department of Internal Medicine II, Medical University of Vienna, Vienna, Austria; 2 Institute of Information Systems Engineering, Research Unit of Information and Software Engineering, Vienna University of Technology, 1040 Vienna, Austria; 3 Department of Internal Medicine I, Klinik Donaustadt, Vienna, Austria; 4 Biobank, Department of Laboratory Medicine, Medical University of Vienna, Vienna, Austria; 5 Department of Laboratory Medicine, Medical University of Vienna, Vienna, Austria; 6 Center for Virology, Medical University of Vienna, Vienna, Austria; 7 Division of Cardiovascular and Interventional Radiology, Department of Biomedical Imaging and Image-guided Therapy, Medical University of Vienna, Vienna, Austria

**Keywords:** Medical research, Health care

## Abstract

Epstein–Barr virus (EBV) reactivation may be involved in long-COVID symptoms, but reactivation of other viruses as a factor has received less attention. Here we evaluated the reactivation of parvovirus-B19 and several members of the Herpesviridae family (DNA viruses) in patients with long-COVID syndrome. We hypothesized that monovalent COVID-19 vaccines inhibit viral interference between SARS-CoV-2 and several DNA viruses in patients with long-COVID syndrome, thereby reducing clinical symptoms. Clinical and laboratory data for 252 consecutive patients with PCR-verified past SARS-CoV-2 infection and long-COVID syndrome (155 vaccinated and 97 non-vaccinated) were recorded during April 2021–May 2022 (median 243 days post-COVID-19 infection). DNA virus–related IgG and IgM titers were compared between vaccinated and non-vaccinated long-COVID patients and with age- and sex-matched non-infected, unvaccinated (pan-negative for spike-antibody) controls. Vaccination with monovalent COVID-19 vaccines was associated with significantly less frequent fatigue and multiorgan symptoms (*p* < 0.001), significantly less cumulative DNA virus–related IgM positivity, significantly lower levels of plasma IgG subfractions 2 and 4, and significantly lower quantitative cytomegalovirus IgG and IgM and EBV IgM titers. These results indicate that anti-SARS-CoV-2 vaccination may interrupt viral cross-talk in patients with long-COVID syndrome (ClinicalTrials.gov Identifier: NCT05398952).

## Introduction

Like other RNA viruses, SARS-CoV-2 can induce a violent cytokine storm and potentially cause persistent infections, as evidenced by the persistence of viral particles in several organs. A lingering viral infection may explain the prolonged symptoms in patients with long-COVID syndrome^1^.

Reactivation of certain viruses—such as hepatitis B, Epstein–Barr virus (EBV), cytomegalovirus (CMV), and herpes simplex virus (HSV)—has been reported among critically ill immunocompetent hospitalized patients, especially in those undergoing immunosuppressive therapy or chemotherapy, reflecting immunosenescence^2–4^. Several clinical investigations among patients hospitalized or treated in the intensive care unit have confirmed co-infections with SARS-CoV-2 and other respiratory viruses, such as influenza and respiratory syncytial virus, or viruses with typical lifelong latency in the nasopharyngeal area (e.g., EBV and rhinoviruses), suggesting that SARS-CoV-2 may reactivate these viruses^5–10^. Anecdotal case reports also have confirmed co-incidence or co-infection of EBV with SARS-CoV-2 in patients with active COVID-19 not requiring hospitalization^11–15^. Some investigations have demonstrated SARS-CoV-2–related EBV reactivation lasting long after the initial COVID-19 illness, suggesting that temporary EBV viraemia may be causative in chronic fatigue syndrome development in the post-acute sequelae or long-COVID phase^16–21^.

In an in vitro study, Verma et al. demonstrated that lytic EBV replication enhances cell surface expression of ACE2 receptors, enabling cellular entry of SARS-CoV-2 and suggesting DNA–RNA inter-viral communication at the cellular level^22^. Interaction between different viruses, involving competitive inhibition or enhancement of viral replication, has been described in children with respiratory tract infections^23^. Virus interference has been investigated during influenza epidemics (i.e., co-circulation of influenza and other respiratory viruses), with a suspected influence on influenza vaccine effectiveness^24^.

Clinical studies have shown a decline in long-COVID symptoms after vaccination, suggesting a protective role of COVID-19 vaccine against persistent subclinical SARS-CoV-2 viraemia^25,26^. However, simultaneous or sequential reactivation of several viruses and the behavior of this “viral consortium” after vaccination targeting a single virus (SARS-CoV-2) have not been investigated in patients with previous SARS-CoV-2 infection.

In this prospective multicenter study, we tested several hypotheses using data from our patients with long-COVID. Our main hypothesis was that COVID-19 vaccination overall, before or after infection, would be linked to decreased SARS-CoV-2–associated reactivation of several DNA viruses of the Herpesviridae family, including HSV, varicella-zoster virus (VZV), CMV, and EBV, as well as parvovirus-B19, in parallel with improvement in long-COVID symptoms from infections during Delta and Omicron SARS-CoV-2 variant predominance. Second, we used data from a subgroup of patients vaccinated before SARS-CoV-2 infection to test our hypothesis that COVID-19 vaccination before infection would be protective compared with after infection, and dampen elevations in plasma DNA virus–related IgG and IgM. Finally, we tested our prediction that plasma levels of DNA viral antibody titers would be higher in long-COVID patients compared with healthy unvaccinated, non-infected controls (i.e., pan-negative for spike-antibody controls).

## Results

**Fig. 1:**
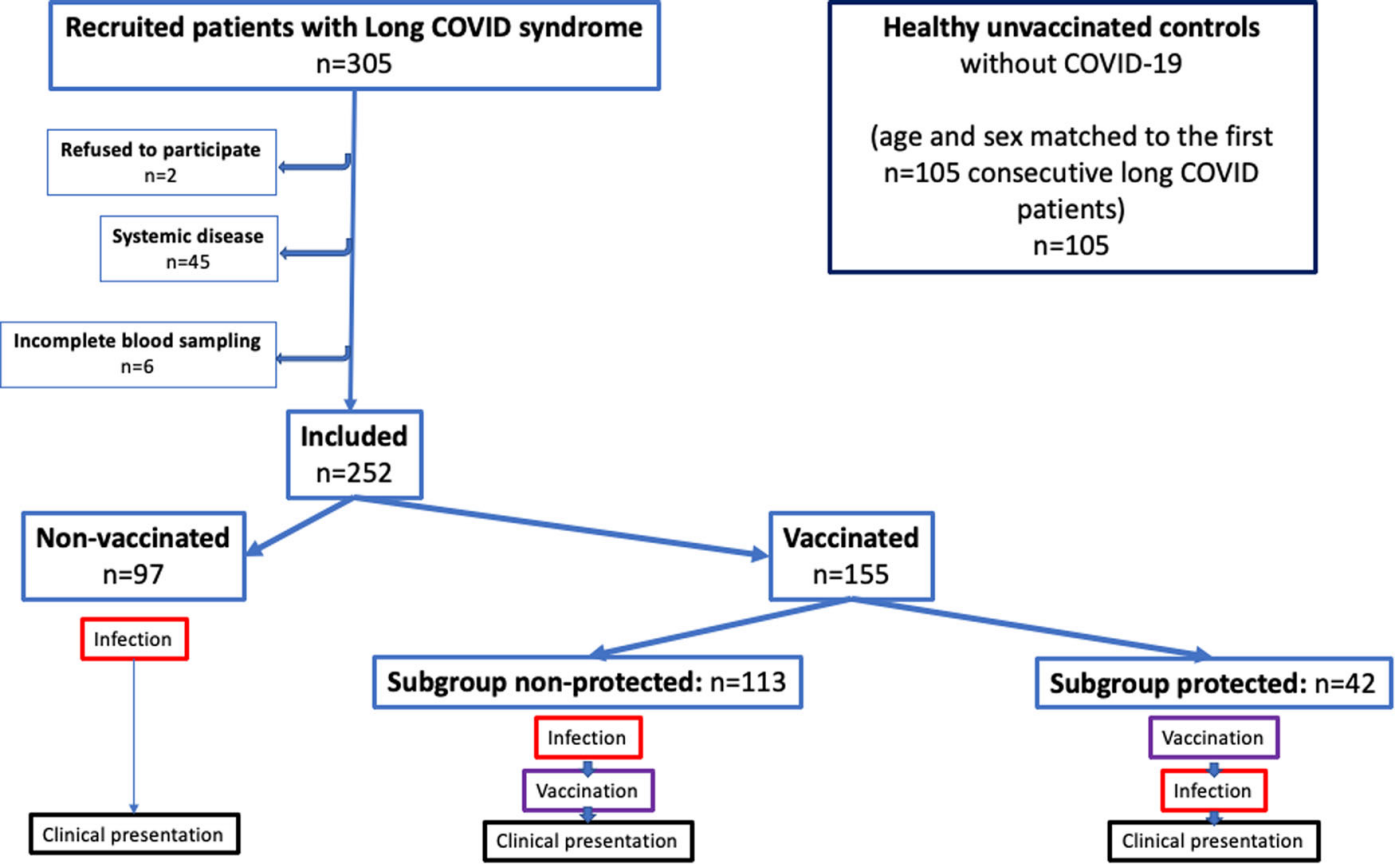
Flowchart of the study. Total 252 patients were prospectively included (left panel). Data of the first 105 patients were compared with healthy unvaccinated controls (right upper panel).

From April 2021 to May 2022, a total of 305 consecutive patients with persistent long-COVID symptoms meeting the criteria for long-COVID syndrome^27–30^ (Supplementary Fig. 1) were prospectively entered into our registry at the Cardiology Long-COVID Unit of the Division of Cardiology, Department of Internal Medicine II, Medical University of Vienna, Austria (EK: 1008/2021 and 1758/2022); ClinicalTrials.gov Identifier: NCT05398952). After exclusion of 53 patients, 252 patients were included in the current analysis (Fig. 1). Reasons for exclusion were refusal to participate (*n* = 2), known systemic inflammatory or active malignant diseases (*n* = 17), newly diagnosed systemic disease (e.g., active hyper- or hypothyroidism, malignant hematologic disorder, acute pulmonary embolism, or ischemic heart disease requiring invasive treatment; *n* = 21), repeated SARS-CoV-2 infection within 3 months before the clinical presentation (*n* = 7), or incomplete blood sampling for any reason (*n* = 6).

The vast majority of patients had an infection during the fourth surge of the COVID pandemic, presumably involving Delta (B.1.617.2 or AY.4.2) variants. Only 20 of the 252 patients (7.9%) were infected with an Omicron SARS-CoV-2 variant (BA.2.12.1), 17 of whom were fully vaccinated and 3 had refused vaccination for any reason (Supplementary Table 1).

Of these 252 patients with long-COVID syndrome, at the time of presentation to our clinic, 155 had been vaccinated either before or after their SARS-CoV-2 infection (vaccinated), and 97 were non-vaccinated, for any reason before presenting; of this latter group, 34 patients received vaccination after the first blood sampling. As indicated in Fig. 1, 113 of the 155 vaccinated patients had COVID-19 before receiving a first vaccine (“subgroup non-protected”), and 42 were vaccinated before infection with SARS-CoV-2 (i.e., “subgroup protected”).

### Vaccination coverage and SARS-CoV-2 reinfection

The mean number of vaccines received was 1.8 ± 0.7, and all administered vaccines were monovalent.

Of the 155 vaccinated patients presenting to our clinic, 65 had received a second dose of vaccine, and 4 had received only a single dose of the Janssen vaccine, so 69 of 155 patients (44.5%) were considered fully immunized (per definition) by the time they presented with long-COVID and underwent blood sampling.

In accordance with the rules during 2021–2022, patients received a single mRNA vaccine dose at 6–9 months after the SARS-CoV-2 infection, which explains, on the one hand, the relatively high number of non-vaccinated patients, and on the other hand, the high proportion of a single vaccine. Only eight patients in our cohort refused vaccination for personal reasons, such as fear of vaccine-induced complications.

Of the 155 vaccinated patients, 34 (21.9%) received a booster (i.e., a third vaccine) before the blood sampling at the first clinical presentation, all of which were a monovalent mRNA vaccine (Supplementary Fig. 2).

Of the 155 vaccinated patients, three (1.9%) had COVID-19 twice. Two of them had received one vaccine dose and one had received two vaccine doses before the second infection. All three received the Comirnaty mRNA vaccine (Pfizer/BioNTech).

Of the 97 patients who were non-vaccinated at presentation, nine (9.3%) were reinfected by SARS-CoV-2. Of note, the clinical presentation and blood sampling in most of these patients were performed before the vaccine rollout.

A total of 131 patients underwent a second blood sampling. Of these, 72 were in the vaccinated group, and 25 remained non-vaccinated at the second sampling. Another 34 patients who were non-vaccinated at presentation later received a vaccine after their initial blood sampling for long-COVID and then had a second blood sampling.

### Main hypothesis: anti-SARS-CoV-2 vaccine mitigates long-COVID syndrome and counteracts DNA virus reactivation

Table 1 presents the clinical data for all included patients with long-COVID (*n* = 252). The results show a significant difference in plasma anti-spike protein titers between vaccinated compared with non-vaccinated patients.

**Table 1:** Clinical data of the included patients.

Clinical data	All patients (*n* = 252)	Patients without vaccination (*n* = 97; 38%)	Patients already vaccinated before the first clinical presentation (*n* = 155; 62%)	*p* between w/wo vaccine
Gender female	170 (67.5%)	66 (68.0%)	104 (67.1%)	
Age years	43.7 ± 14.2	43.3 ± 13.1	43.9 ± 14.9	
DM	7 (2.8%)	1 (1.0%)	6 (3.9%)	
Hypertension	70 (27.8%)	22 (22.7%)	48 (31.0%)	
HLP	59 (23.4%)	20 (20.6%)	39 (25.2%)	
Smoking	22 (8.7%)	6 (6.2%)	16 (10.3%)	
Syst RR mmHg	131 ± 17	130 ± 16	132 ± 17	
RR diast mmHg	84 ± 11	84 ± 10	83 ± 11	
Heart rate (bpm)	72 ± 12	71 ± 11	72 ± 12	
Patient category				
1 (Neuro)	99 (39.3%)	38 (38%)	61 (39%)	
2 (Pulmo)	59 (23.4%)	21 (22%)	38 (25%)	
3 (Cardio)	94 (37.3%)	38 (39%)	56 (36%)	
COVID-related data				
Time between COVID-19 positivity and first clinical presentation (days) (median; IQR)	243 (139; 360)	190 (130; 275)	278 (143; 388)	**0.001**
Time between COVID vaccine and first clinical presentation (days)			173 (77; 300)	
Anti-spike protein titer BAU/mL	2272 (133; 2500)	137 (35; 623)	2500 (2500; 2500)	<**0.001**
ECG				
Any ECG Abnormalities	61 (24.2%)	19 (19.6%)	42 (27.1%)	
Rhythm disturbances	14 (5.6%)	6 (6.2%)	8 (5.2%)	
Conduction abnormalities	52 (20.6%)	14 (14.4%)	38 (24.5%)	
QRS duration (ms)	92 ± 14	94 ± 15	91 ± 14	

Data are presented as mean ± SD, median (interquartile range), or number (frequency).

Statistical comparisons between median values of relevant parameters of both groups were performed using the two-sided non-parametric Mann–Whitney *U* test.

Bold values represent significant differences between patients groups w/wo vaccination.

**Fig. 2:**
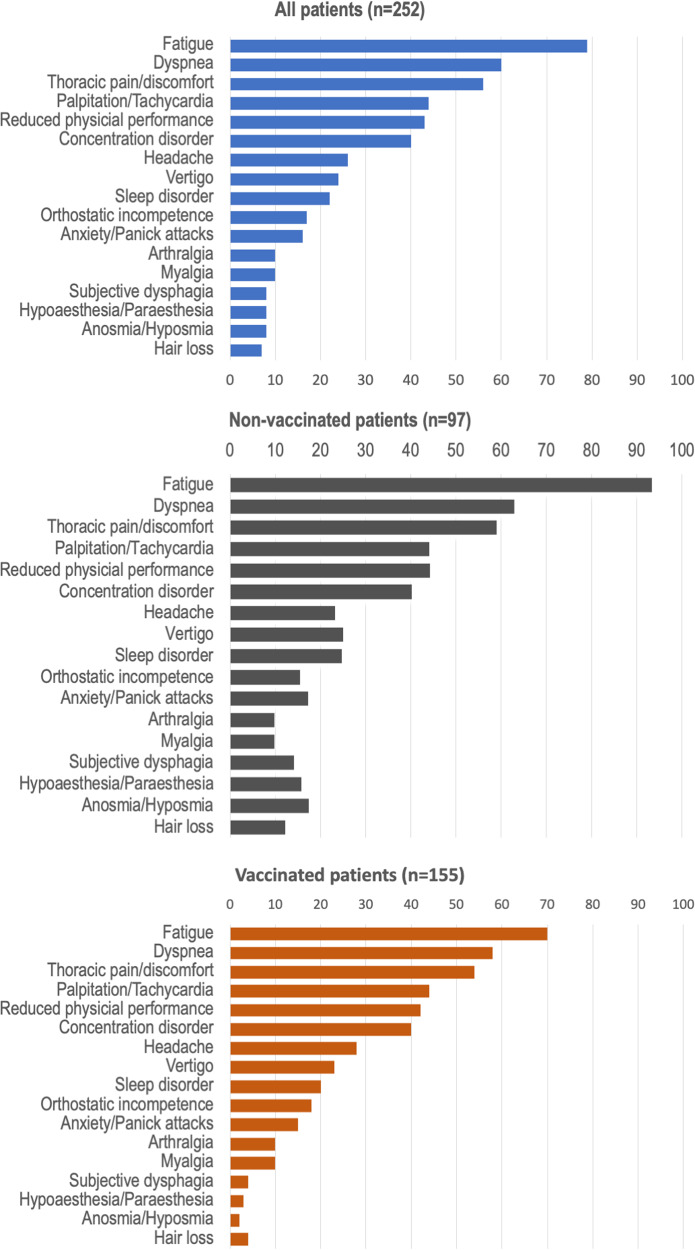
Clinical symptoms at the first clinical presentation. Clinical complaints of all patients (upper panel), non-vaccinated (mid panel) or vaccinated patients (bottom panel) at the first clinical presentation.

The most common symptoms were neuropsychiatric (without objective pulmonary or cardiovascular abnormalities), followed by predominantly cardiovascular symptoms and pulmonary diseases (Fig. 2). Compared with non-vaccinated patients, vaccinated patients had fatigue significantly less frequently (*p* < 0.001) and less often had a combination of several (≥3) multiorgan symptoms (*p* < 0.001). (Comparison between the non-vaccinated versus vaccinated patients was calculated by chi-quadrat test.)

Table 2 shows the clinical laboratory data. The vaccinated and non-vaccinated patients did not differ significantly in any of these measures. No conspicuous pathologic laboratory results were reported for any patients. The vast majority of circulating biomarkers remained in the normal range, including coagulation, hematologic, and cardiologic parameters. Marginally elevated laboratory values were followed up with thorough clinical investigations to exclude organ diseases. Acute pulmonary embolism was excluded in patients with mildly elevated D-dimer (all <1.0 ug/mL) and age ≤50 years, and additional mild obstructive pulmonary disease was diagnosed in two patients.

**Table 2:** Clinical laboratory data showing no difference between vaccinated and non-vaccinated patients.

Routine clinical lab data	All patients (*n* = 252)	Patients without vaccination (*n* = 97; 38%)	Patients already vaccinated before the first clinical presentation (*n* = 155; 62%)
Hematology and general			
Hgb, g/dL	14.0 ± 1.3	13.9 ± 1.3	14.1 ± 1.3
Platelet, G/L	256 ± 55	258 ± 54	254 ± 56
Leukocyte, G/L	6.6 ± 1.8	6.6 ± 1.9	6.5 ± 1.7
Creatinin, mg/dL	0.78 ± 0.15	0.78 ± 0.17	0.77 ± 0.14
Albumin, g/L	47.5 ± 2.8	47.5 ± 2.7	47.5 ± 2.9
SGOT, U/L	24 ± 10	25 ± 12	24 ± 9
SGPT, U/L	28 ± 23	30 ± 25	27 ± 22
Eisen, ug/dL	92 ± 35.1	92.1 ± 38.8	91.9 ± 32.8
TSH, u/U/mL	1.58 ± 0.93	1.57 ± 0.8	1.59 ± 1
Coagulation			
Prothrombin time, %	98.7 ± 18.4	97.2 ± 17.3	99.6 ± 19.1
INR	1.1 ± 0.2	1.0 ± 0.1	1.1 ± 0.2
aPTT, s	35.3 ± 4	35.7 ± 4.5	35.1 ± 3.7
Fibrinogen, mg/dL	312 ± 66.8	307 ± 68	315 ± 66
D-dimer, ug/mL	0 (0; 0.39)	0 (0; 0.35)	0.14 (0; 0.41)
Elevated D-dimer	18/223 (8.1%)^a^	6/88 (6.8%)	12/135 (8.9%)
vWF antigen, %	125 ± 53	129 ± 46	123 ± 57
ADAMTs13 activity, %	107 ± 27	108 ± 28	106 ± 26
Cardiology			
Troponin T, ng/L	0 (0; 5)	0 (0; 5)	0 (0; 5.25)
Elevated troponin T	6/240 (2.5%)^b^	3/93 (3.2%)	3/149 (2.0%)
Creatine kinase, U/L	106 ± 66	103 ± 68	107 ± 65
NT-proBNP, pg/mL	45.4 (28.5; 85.1)	46.4 (26.3; 80.4)	45.4 (29.9; 92.8)
Elevated proBNP	15/243 (6.2%)^c^	5/92 (5.4%)	10/151 (6.6%)

Data are presented as mean ± SD, median (interquartile range), or number (frequency).

^a^Two patients were diagnosed with mild chronic obstructive pulmonary disease.

^b^Acute coronary syndrome was excluded in all patients.

^c^Patients were thoroughly evaluated for cardiac and pulmonary disease.

Table 3 presents circulating inflammatory biomarker levels. Acute infection was excluded in all patients. Mean and median values of the inflammatory biomarkers remained in the normal range. Compared with non-vaccinated patients, vaccinated patients showed a trend towards lower total IgG values, with significantly lower levels of IgG subfractions 2 and 4.

**Table 3:** Circulating inflammatory biomarker levels.

Inflammatory parameter	All patients (*n* = 252)	Patients without vaccination (*n* = 97; 38%)	Patients already vaccinated before the first clinical presentation (*n* = 155; 62%)	*p* between w/wo vaccine
C-reactive protein, mg/dL	0.09 (0.05; 0.21)	0.09 (0.04 ;0.18)	0.11 (0.05; 0.23)	
LDH, U/L	166 (153; 186)	166 (151; 186)	167 (156; 186)	
Ferritin, ug/L	79.2 (40.1; 151)	66.8 (33; 151.8)	90.4 (46.4; 152.7)	
Transferrin, mg/dL	269 ± 42	264 ± 37	271 ± 45	
Transferrin saturation, %	25.2 ± 11.4	25.4 ± 11.6	25.0 ± 11.3	
Histamin, mg/dL	7.3 ± 2.6	7.0 ± 3.7	7.5 ± 3.5	
IL-6, pg/mL	1.57 (0; 2.26)	1.57 (0; 2.34)	1.56 (0; 2.2)	
Procalcitonin, ng/mL	0.03 (0; 0.04)	0.03 (0; 0.04)	0.03 (0; 0.04)	
Total IgG, mg/dL	1125 ± 283	1168 ± 355	1098 ± 224	
Total IgA, mg/dL	204 ± 84	212 ± 87	199 ± 82	
Total IgM, mg/dL	107 ± 51	108 ± 63	107 ± 50	
Total IgE, kIU/L	27.7 (11.7; 78.4)	33.6 (10.9; 86.8)	26 (12.9; 74.1)	
IgG1 subfraction, mg/dL	700 ± 191	721 ± 241	688 ± 151	
IgG2 subfraction, mg/dL	347 ± 139	373 ± 165	331 ± 118	**0.026**
IgG3 subfraction, mg/dL	37 ± 21	38.2 ± 24.4	36.3 ± 17.8	
IgG4 subfraction, mg/dL	61 ± 56	72.2 ± 68.8	53.3 ± 44.4	**0.014**
Tryptase, ug/L	4.8 ± 2.8	4.7 ± 2.7	4.8 ± 2.9	
Rheumafactor latex, IU/mL	0 (0; 0)	0 (0; 0)	0 (0; 0)	
Alpha 1 antitrypsin, mg/dL	136 ± 23	137 ± 21	135 ± 25	
Cardiolipin IgG, U/mL	1.3 (1.1; 1.7)	1.3 (1.1; 1.7)	1.3 (1.1; 1.7)	
Cardiolipin IgM, U/mL	1.4 (1; 2.25)	1.4 (0; 2)	2.4 (1; 2.3)	

Data are presented as mean ± SD or median (interquartile range).

Differences between groups were calculated using the two-sided Student’s *t*-test with Holm–Bonferroni corrections.

Bold values represent significant differences between patients groups w/wo vaccination.

Table 4 presents the qualitative and quantitative plasma virus titers in all patients and in the vaccinated and non-vaccinated groups. Among all patients, 15.1% presented cumulative IgM positivity or an elevated virus-specific polymerase chain reaction (PCR) level, as did 21.6% of non-vaccinated patients versus 11% of vaccinated patients (*p* = 0.029). Among all patients, 34.4% had higher EBV nuclear antigen IgG titers, and 36.3% had higher HSV nuclear antigen IgG titers, above the detection limit. This finding could be interpreted as reactivations of EBV or HSV infection, as supported by the current literature^19^. Vaccination was associated with significantly lower cumulative IgM positivity of the investigated DNA viruses and with lower CMV IgG, CMV IgM, and EBV IgM. This finding suggested a vaccine-induced decrease in antibody production triggered by virus–virus interaction. Of interest, the parvovirus-B19 IgG titer was increased in the vaccinated group.

**Table 4:** Peripheral blood qualitative and quantitative IgG and IgM virus titers.

Routine virology lab data	All patients (*n* = 252)	Patients without vaccination (*n* = 97; 38%)	Patients already vaccinated before the first clinical presentation *(n* = 155; 62%)	*p* between w/wo vaccine
Quantitative data				
CMV IgG, mg/dL	62.3 (0; 116)	87.3 (0; 119.0)	11.4 (0; 114)	**0.044**
CMV IgM, mg/dL	0 (0; 0)	0 (0; 5.9)	0 (0; 0)	**0.004**
EBV IgG, mg/dL	310 (105; 750)	402 (136; 750)	267 (99; 750)	
EBV IgM, mg/dL	0 (0; 0)	0 (0; 8.53)	0 (0; 0)	**0.025**
EBV EBNA IgG, mg/dL	242 (66; 516)	272 (70; 529)	220 (65; 514)	
HSV IgG, mg/dL	22.2 (0.9; 30.0)	22.8 (1.0; 30)	22.1 (0.8; 30)	
HSV IgM, mg/dL	0 (0; 0)	0 (0; 5.1)	0 (0; 0)	
VZV IgG, mg/dL	1076 (661; 1577)	1108 (661; 1567)	1068 (652; 1584)	
VZV IgM, mg/dL	0.19 (0.14; 0.28)	0.22 (0.16; 0.3)	0.18 (0.14; 0.27)	
Parvo-B19 IgG, mg/dL	22 (2.4; 43.0)	16 (0; 39)	27 (3.4; 46)	**0.028**
Parvo-B19 IgM, mg/dL	0.20 (0; 0.45)	0.25 (0; 0.47)	0 (0.05; 0.41)	0.056
Qualitative data				
Cumulative virus IgM positivity (*n* = 252)	38 (15.1%)	21 (21.6%)	17 (11.0%)	**0.029**
CMV IgG positivity (*n* = 235)	144 (61.3%)	59 (71.1%)	85 (55.9%)	**0.025**
CMV IgM positivity (*n* = 235)	8 (3.4%)	4 (4.8%)	4 (2.6%)	
EBV IgG positivity (*n* = 244)	236 (96.7%)	91 (97.8%)	145 (96.0%)	
EBV IgG positivity above detection limit (*n* = 244)	84 (34.4%)	37 (39.8%)	47 (31.1%)	
EBV IgM positivity (*n* = 244)	10 (4.1%)	7 (7.5%)	3 (2.0%)	**0.046**
EBV EBNA IgG positivity (*n* = 244)	213 (87.3%)	83 (89.2%)	130 (86.1%)	
EBV EBNA IgG above detection limit (*n* = 244)	52 (21.3%)	20 (21.5%)	32 (21.2%)	
HSV IgG positivity (*n* = 237)	211 (89.0%)	79 (92.9%)	132 (86.8%)	
HSV IgG positivity above detection limit (*n* = 237)	86 (36.3%)	34 (41.0%)	52 (34.2%)	
HSV IgM positivity (*n* = 235)	8 (3.4%)	3 (3.7%)	5 (3.3%)	
VZV IgG positivity (*n* = 235)	235 (100%)	82 (100%)	153 (100%)	
VZV IgM positivity (*n* = 235)	2 (0.8%)	0 (0%)	2 (1.3%)	
Parvo-B19 IgG positivity (*n* = 247)	195 (78.9%)	71 (74.5%)	124 (81.6%)	
Parvo-B19 IgM positivity (*n* = 247)	15 (6.1%)	7 (7.4%)	8 (5.3%)	

Data are presented as mean ± SD, median (interquartile ranges), or number (frequency).

Statistical comparisons between the groups were calculated by two-sided non-parametric Mann–Whitney *U* test for data with a non-normal distribution and the *χ*^2^ test for nominal variables.

Bold values represent significant differences between patients groups w/wo vaccination.

**Fig. 3:**
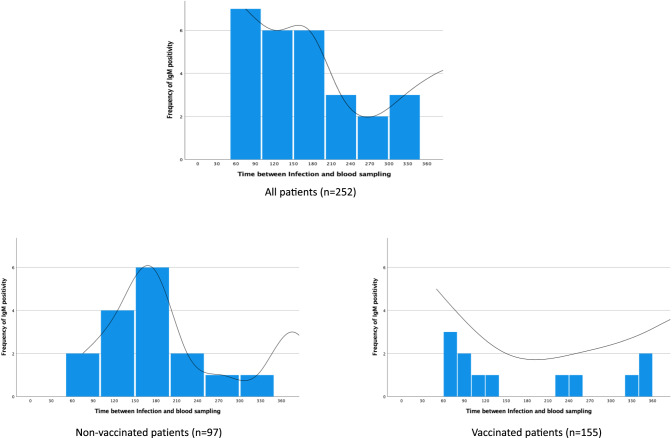
Cumulative IgM positivity, including herpes simplex virus (HSV), varicella-zoster virus (VZV), cytomegalovirus (CMV), Epstein–Barr virus (EBV), and parvovirus-B19. Time-dependent cumulative IgM positivity in all patients with long-COVID syndrome (upper panel), non-vaccinated patients (bottom left panel) and vaccinated patients (bottom right panel) at the first clinical presentation.

In contrast with the vaccinated population, non-vaccinated patients showed a temporary increase in cumulative IgM virus positivity (Fig. 3).

**Fig. 4:**
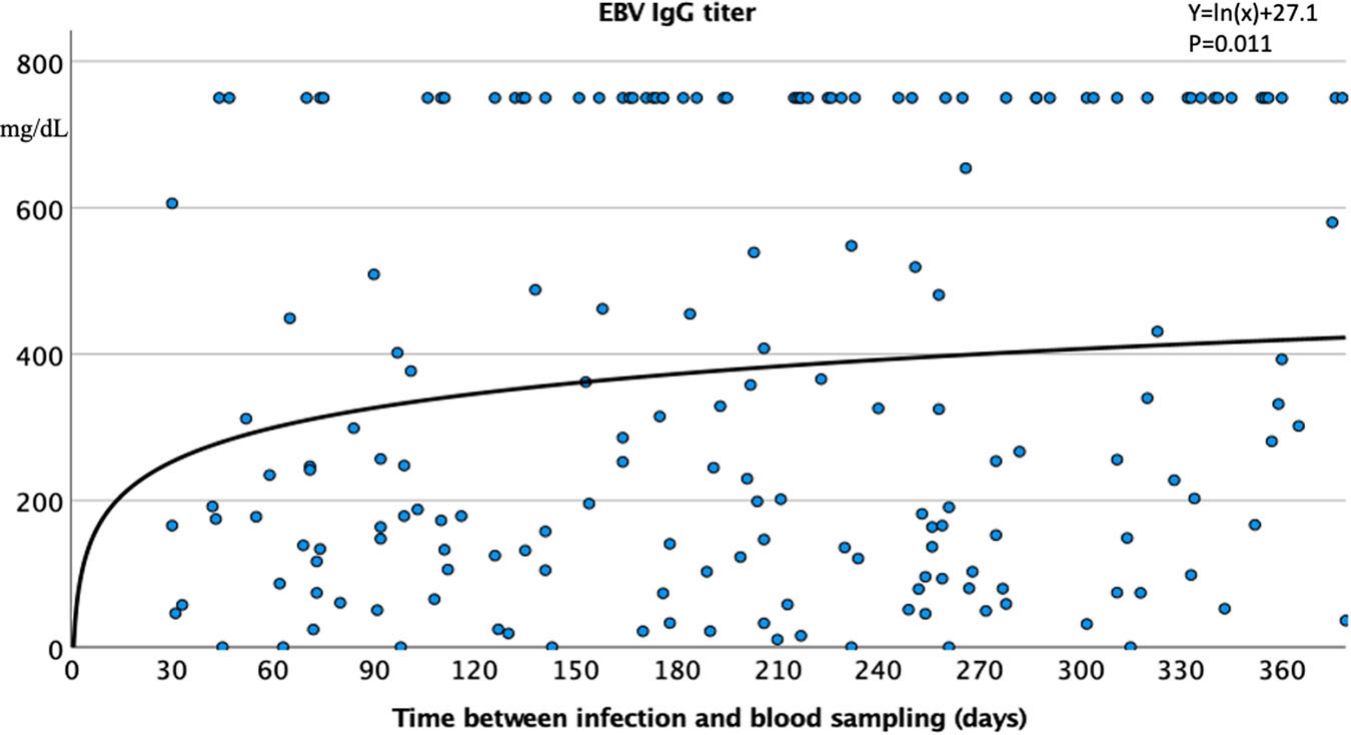
Quantitative Epstein–Barr virus (EBV) IgG titer in the full cohort with long-COVID (*n* = 252; detection limit: 750 mg/dL). Time-dependent increase in the EBV IgG titer after SARS-CoV-2 infection.

**Fig. 5:**
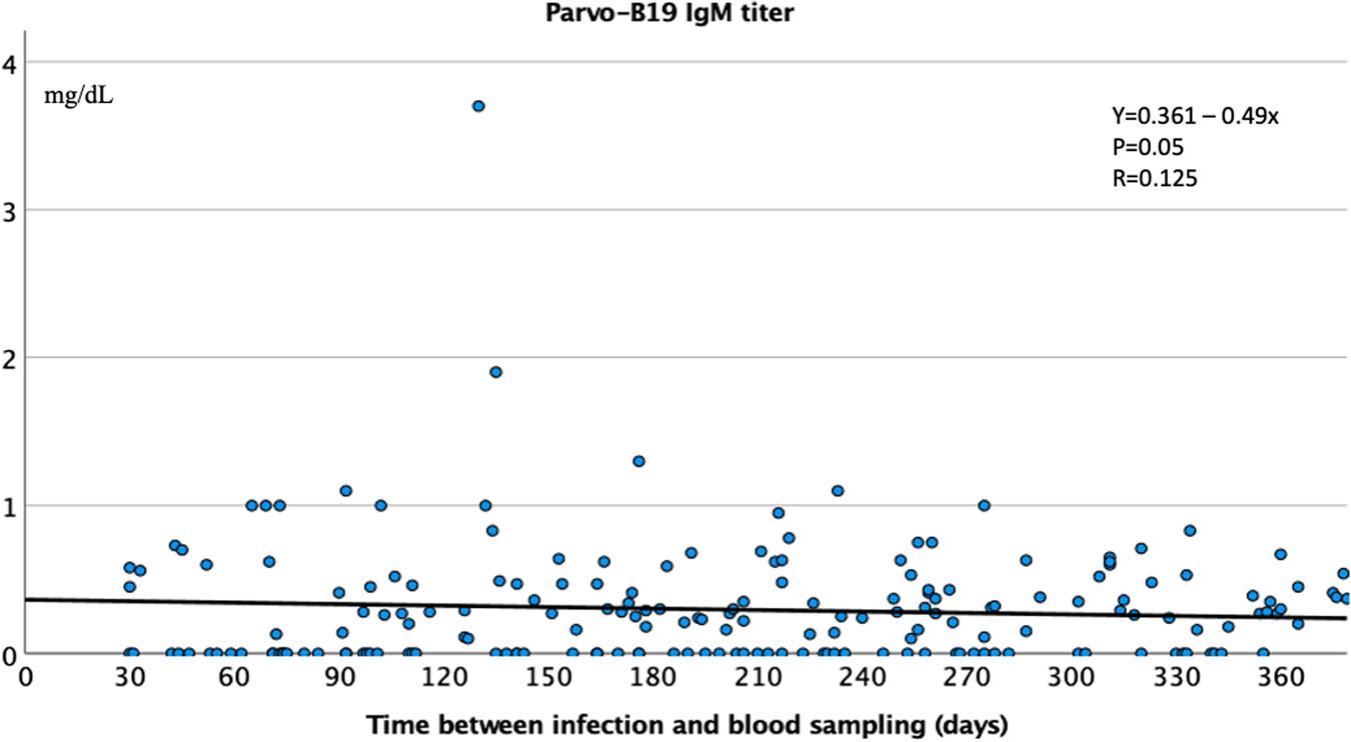
Quantitative parvovirus-B19 IgM titer in the overall cohort with long-COVID (*n* = 247). Time-dependent decrease in the parvovirus-B19 IgM titer after SARS-CoV-2 infection.

We observed a significant logarithmic correlation between time to infection and quantitative EBV IgG titer, showing an increase in EBV IgG titer over time (Fig. 4). Vaccination only marginally influenced this increase (Table 4). In contrast, the quantitative titer of parvovirus-B19 IgM exhibited a linear decrease (Fig. 5).

Other lab values showed no time-dependent changes, including hematologic, coagulation, and inflammatory biomarkers, routine lab measurements (e.g., kidney, liver, cardiac), and viral titers at the first clinical presentation.

DNA virus IgG and IgM titers did not differ between groups with dominant neuropsychiatric, pulmonary, or cardiologic symptoms, except in two instances. First, 47.4% of patients in the pulmonary group had an EBV VCA IgG titer over the detection limit, compared with 32.2% in the neuropsychiatric group and 18.5% in the cardiologic group (*p* = 0.013). Second, 90.9% of patients in the cardiologic group had parvovirus-B19 IgG positivity, compared with 74.2% in the neuropsychiatric group and 84.2% in the pulmonary group (*p* = 0.022, for comparisons between the subgroups, *χ*^2^ test were performed).

### Second hypothesis: anti-SARS-CoV-2 vaccination is protective in patients who received the vaccine before a SARS-CoV-2 infection

Supplementary Tables 2–5 present the clinical and laboratory data for the subgroup of patients who received the vaccine before they had COVID-19 (“subgroup protected”), compared with patients who had a SARS-CoV-2 infection before vaccination (“subgroup non-protected”). Patients in the “protected” group had significantly lower NT-proBNP and cardiolipin IgM. Immunoprotection before SARS-CoV-2 infection was associated with significantly lower EBV IgG, a lower incidence of EBV IgG positivity, and a lower frequency of high EBV VCA IgG over the detection limit (Supplementary Table 5).

### Third hypothesis: vaccination reduces plasma DNA virus IgG and IgM in patients with repeated clinical presentation and blood sampling

A total of 131 patients had a second clinical assessment with blood sampling, with an interval of 106 ± 77 days between baseline and follow-up blood sampling times. Clinical laboratory values did not differ between the first and second samplings. Among these patients, from the first to the second blood sampling time, we observed significant decreases in cumulative virus IgM positivity (from 19.8% to 11.5%, *p* = 0.044, *χ*^2^ test) and in parvovirus-B19 IgM titers (from 0.45 ± 1.7 to 0.21 ± 0.32 mg/dL, *p* = 0.019, two-sided Student’s *t*-test).

Of these 131 patients, 72 were vaccinated at presentation. A total of 34 of 97 non-vaccinated patients at presentation later received a vaccination between the first and second blood samplings (post-presentation vaccinated) and were also analyzed. Considering that vaccination influenced viral IgG and IgM titers (see main study findings above), the vaccinations received before and after the first blood sampling might introduce bias into the interpretation of these results.

Finally, among the 131 patients who had a second sampling were 25 non-vaccinated patients who remained unvaccinated. In this group, the only significant difference between the first and second blood samplings was an increase in parvovirus-B19 IgG positivity from 68% to 100% (*p* = 0.004, *χ*^2^ test).

### Fourth hypothesis: long-COVID patients have higher plasma DNA viral antibody titers than healthy unvaccinated, non-infected controls (pan-negative to anti-SARS-CoV-2 antibody)

For the first 105 consecutive patients with long-COVID syndrome (age 46 ± 15 years, 36.2% male), clinical data and blood samples were collected from March 15, 2021, to September 30, 2021. Blood samples of age- and sex-matched (46 ± 12 years, 36.2% male) healthy, unvaccinated, non-infected individuals collected from June 18, 2020, to November 11, 2020 (EC: 1387/2020; ClinicalTrials.gov Identifier: NCT04407429)^31^ were retrieved from the biobank facility of the Medical University of Vienna (with processing and storage in accordance with the standard operating procedures and an ISO 9001:2015)^32^. Information on sex and age was obtained through the hospital’s electronic database. These individuals were not yet vaccinated and had no spike protein or nucleocapsid antibodies, indicating no previous SARS-CoV-2 infection.

For all long-COVID patients, the time between SARS-CoV-2 infection and their first clinical visit was 219 ± 98 days (7 ± 3 months). Anti-spike protein antibody was zero in healthy controls, and 1162.6 ± 1150.7 BAU/mL among all long-COVID patients.

**Fig. 6:**
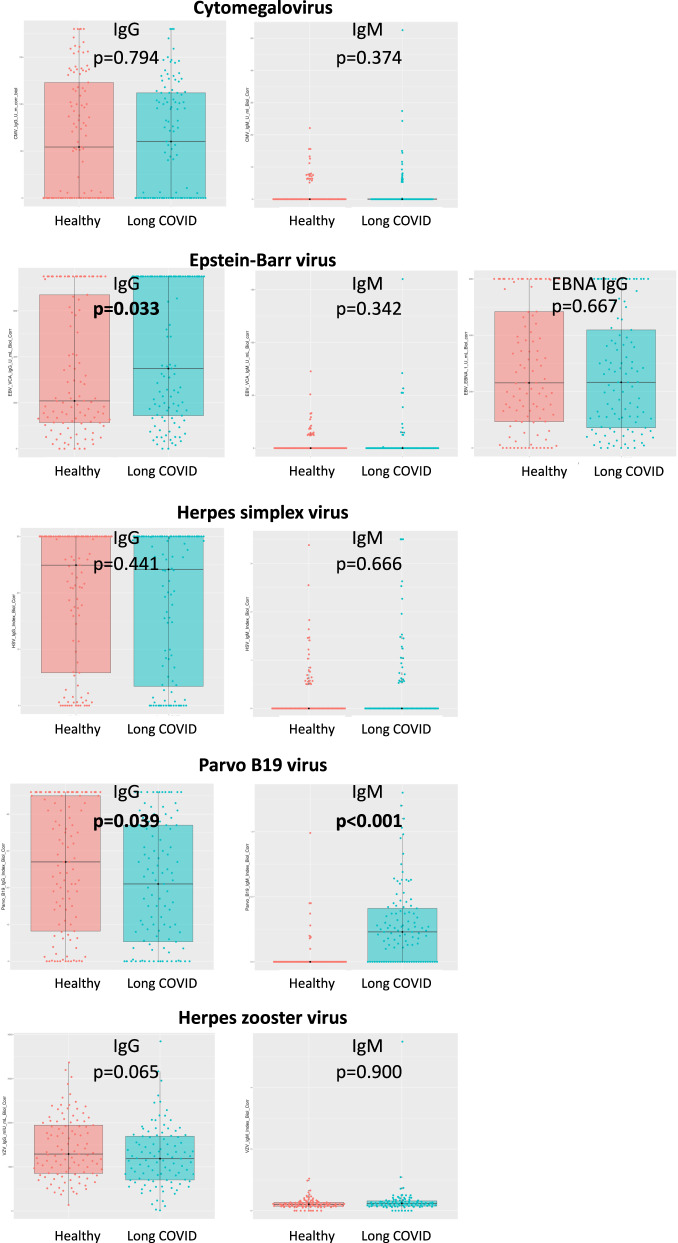
IgG and IgM virus titers (mg/dL) among patients with long-COVID (*n* = 105) and age- and sex-matched healthy controls (*n* = 105). Box plots with median values (center line), first and third quantiles (shaded box), minimum and maximum values (horizontal lines). Differences between groups were calculated using the two-sided non-parametric Mann–Whitney *U* test.

Table 5 shows the qualitative results. Figure 6 presents the box plots of the quantitative IgG and IgM virus titers of the investigated DNA viruses, revealing significantly higher EBV VCA IgG titers in long-COVID patients compared with healthy controls (*p* = 0.033). Of interest, the long-COVID patients had a significantly lower parvovirus-B19 IgG titer but a significantly higher IgM titer (*p* < 0.001) (Fig. 6 and Table 5) (Differences between groups were calculated using the two-sided non-parametric Mann–Whitney *U* test).

**Table 5:** Qualitative IgG and IgM titers of the investigated viruses among patients with long-COVID syndrome and healthy (non-infected, unvaccinated) controls.

	Healthy (*n* = 105)	Long-COVID (*n* = 105)	*p* value
Male	38 (36.2%)	38 (36.2%)	
Cumulative virus IgM positivity	7 (6.7%)	19 (18.1%)	**0.02**
CMV IgG positivity	60 (57.1%)	59/103 (57.3%)	
CMV IgM positivity	1 (1%)	4/103 (3.9%)	
EBV IgG positivity	100 (95.2%)	103 (98.1)	
EBV VCA IgG positivity above detection limit	25 (23.8%)	42 (40.0%)	**0.018**
EBV IgM positivity	2 (1.9%)	7 (6.7%)	
EBV EBNA IgG positivity	91 (86.7%)	100 (95.2%)	
EBV EBNA IgG above detection limit	21 (20%)	18 (17.1%)	
HSV IgG positivity	87 (82.9%)	82/104 (78.8%)	
HSV IgG positivity above detection limit	44 (41.9%)	40/104 (38.5%)	
HSV IgM positivity	2 (1.9%)	6/104 (5.8%)	
VZV IgG positivity	105 (100%)	98/101 (97%)	
VZV IgM positivity	2 (1.9%)	1/101 (1%)	
Parvo_B19 IgG positivity	91 (86.7%)	86 (81.9%	
Parvo_B19 IgG positivity above detection limit	24 (22.9%)	14 (13.3%)	
Parvo_B19 IgM positivity	1 (1%)	6 (5.7%)	

Comparisons were performed by using a two-sided *χ*^2^ test.

Bold values represent significant differences between healthy and long-COVID patient groups.

## Discussion

A main finding of this study of patients with long-COVID syndrome is that SARS-CoV-2 infection apparently activated certain types of DNA viruses (EBV, HSV, CVM, and parvovirus-B19). This activation was indicated by the significantly higher incidence of cumulative IgM positivity and elevated EBV VCA IgG and parvovirus-B19 IgM titers in long-COVID patients compared with healthy controls. Overall, 34.4% and 36.3% of patients, respectively, presented with higher EBV and HSV nuclear antigen IgG titers, over the detection limit of commercially available laboratory tests. The time to infection showed a significant logarithmic correlation with quantitative EBV IgG titer, with the EBV IgG titer increasing over time after SARS-CoV-2 infection. In contrast, the parvovirus-B19 IgM quantitative titer decreased linearly with increasing time after COVID-19.

Another main finding of our study is that anti-SARS-CoV-2 vaccination played a protective role against DNA virus activations (EBV, HSV, CVM, and parvovirus-B19), as demonstrated at the patient level. In detail, compared with long-COVID patients non-vaccinated at presentation, patients who were vaccinated against SARS-CoV-2 at presentation had significantly less frequent fatigue and multiorgan symptoms, significantly lower plasma levels of IgG subfractions 2 and 4, significantly less frequent cumulative IgM positivity or positive virus-specific PCR titer, and significantly lower quantitative CMV IgG, CMV IgM, and EBV IgM titers. Moreover, among vaccinated patients, those who were already immunoprotected against SARS-CoV-2 before their first SARS-CoV-2 infection (“protected subgroup”) had significantly lower EBV VCA IgG titers, lower NT-proBNP plasma levels, and lower cardiolipin IgM titers compared with the “non-protected” group.

Several previous studies have reported co-detection of different viruses (mainly respiratory viruses) in severely ill patients hospitalized with COVID-19^33^. However, approximately 92% of COVID-19 patients with mild or moderate symptoms remained at home, without any medical records being established. No information is available for this population about possible co-infections with viruses or other pathogens that might affect long-term outcomes and development of long-COVID syndrome^34^. The long-COVID phase lasts several months or even years and can include subclinical multiorgan symptoms of variable degrees, which is not typical for an acute infection. Therefore, routine clinical investigations for current pathogen infection are not clinically justified. However, some long-COVID symptoms resemble subclinical post-viral syndromes, such as chronic fatigue syndrome, low-grade fever, rapid exhaustion, and post-exertional malaise. Several groups have suggested that these long-lasting symptoms may be caused by the sequential and prolonged subclinical activation of viruses that are normally co-localized in the nasopharyngeal space^16–21^.

Peluso et al. reported the effect of pre-existing or chronic viral load and reactivation of EBV and CMV on neurocognitive and fatigue symptoms at a median of 4 months after COVID-19 and suggested EBV IgG and EBV nuclear antigen as potential biomarkers of EBV reactivation^21^. Moreover, EBV infection and reactivation may induce autoimmune processes that could further explain chronic subclinical inflammation and related symptoms in long-COVID patients^21^. Seroprevalence of CMV depends on several factors, such as subpopulations (ethnicity, sex, age, socioeconomic status, education level), geographic region, and medical risk factors^35^. Similar to others, Naendrup et al. reported activation of CMV and EBV in patients during severe COVID-19 treated in the intensive care unit^36^. Here, we observed that a high proportion of patients had EBV IgG and EBV nuclear antigen titers above the detection limit, with a high seroprevalence of CMV.

Of note, we excluded patients with significant comorbidities, such as HIV infection, and patients who were hospitalized for severe COVID-19. Moreover, we matched non-infected, unvaccinated control patients to our long-COVID patients and investigated the effect of COVID-19 vaccination on titers of several DNA viruses. Although the beneficial effects of vaccination on long-COVID syndrome have been extensively investigated, a protective effect of vaccination against SARS-CoV-2–associated reactivation of other viruses has not previously been reported. In addition, our patient cohort had a relatively long follow-up (median 8 months post-infection), and many patients had elevated and positive IgM titers of the investigated DNA viruses several months after their initial SARS-CoV-2 infection. This pattern raises the question of whether their symptoms resulted from a prolonged virus–virus interaction, or whether the “co-infection” is independent of the initial SARS-CoV-2 infection and represents a new viral infection in patients with altered immune responses after COVID-19. The elevated IgM of diverse viruses after SARS-CoV-2 infection might also indicate an inappropriate activation of the antiviral memory of the immune B and T cells^37^.

Vaccinated patients had lower IgG2 and IgG4 values. IgG variants such as IgG4 and IgG2 are mostly associated with anti-inflammatory immune processes resulting from suppression of the Fc-mediated antibody response^38^. Full vaccination with two doses of mRNA vaccine induces elevation of the IgG subclasses IgG1 and IgG3, while levels of IgG2 and IgG4 are unaffected^38^. However, a third mRNA vaccine (but not other vaccine types) induces a prominent increase in anti-spike IgG2 and IgG4 antibody levels, with the appearance of specific IgG4-switched B cells in the peripheral circulation^39^. We measured serum IgG subtypes with commercially available laboratory methods without investigating peripheral blood mononuclear cell functions, and only 21.9% of our vaccinated patients received the third booster mRNA vaccine (13 after AstraZeneca and 4 after Janssen). Therefore, the association between the diverse types of monovalent vaccines and lower serum levels of IgG subtypes 2 and 4 in long-COVID patients remains to be clarified.

In our study, vaccination against SARS-CoV-2 (an RNA virus) was associated with decreased DNA viral antibody titers, suggesting that anti-SARS-CoV-2 vaccination may interrupt virus–virus communication. Although an exact mechanism can only be speculated, there are possible explanations for how RNA viruses could activate other RNA or DNA viruses^40^ and an anti-RNA virus molecule could de-activate this viral interference. RNA and DNA viruses have different cellular receptors that affect diverse signaling mechanisms within the innate immune response. However, there is cross-talk involving mechanisms of detection of nucleic acids originating from RNA and DNA viruses and downstream regulators. This cross-talk might explain at least in part the amplification of their interactions^40,41^, such as by suppressing the host antiviral reaction and facilitating invasion of co-occurring viruses, leading to parallel de-activation of several co-localized viruses. Contradicting the assumption that SARS-CoV-2 induces reactivation of other viral pathogens, though, Burstein et al. showed in a large-scale study that virus pairs do not act synergistically, and rather mitigate one another’s infective capacity^42^. For example, acute SARS-CoV-2 infection has reportedly attenuated rhinovirus (a rapidly replicating virus) viral load, suggesting competitive consumption of “cellular nutritional resources” and interference between viral pathogens^40,43^. In addition, several investigations of virus interference have indicated a decreased incidence of certain respiratory viral infections during seasonal influenza pandemics. Indeed, vaccination against influenza has been reported to be protective against non-influenza respiratory viruses^44^. Conversely, virus interference may affect monovalent vaccine effectiveness^24^. There is clearly a need for systemic investigations to elucidate the exact mechanisms of virus interference and its importance in terms of long-term morbidity and outcomes for patients with long-COVID syndromes.

In conclusion, the results of our clinical investigation provide the first demonstration of reactivation of several DNA viruses after SARS-CoV-2 infection (viral cross-talk or interference). We further show the interruption of this viral cross-talk by anti-SARS-CoV-2 vaccination in patients with long-COVID syndrome.

## Methods

### Study design and patients

The POSTCOV cohort study is an ongoing multicenter prospective registry, approved by the Ethical Committee of the Medical University of Vienna and was performed in accordance with the Declaration of Helsinki (EC: 1008/2021, and EC: 1758/2022, ClinicalTrials.gov Identifier: NCT05398952). For data control, the study was extended with a case–control addition (EC: 1387/2020). The study complies with all relevant ethical regulations for clinical work with humans. Informed consent was obtained from all patients.

The presentation of the methods and results conforms with the STROBE guidelines^45^.

### Inclusion and exclusion criteria

Inclusion criteria for long-COVID patients were as follows: (1) previous COVID-19 confirmed by quantitative real-time PCR; (2) previous mild or moderate COVID-19 not requiring hospitalization; (3) absence of previous or present inflammatory disease, malignancies, or chronic organ disorders (e.g., renal insufficiency, chronic heart or lung disease, or rheumatic diseases); and (4) at least three symptoms from three different organs fulfilling the criteria of long-COVID syndrome. Exclusion criteria were as follows: (1) clinically confirmed active infection combined with elevated inflammatory markers, such as C-reactive protein, leukocytes, and fibrinogen; (2) no verified past SARS-CoV-2 infection, or missing PCR test; (3) clinically acute infection of any kind independent from laboratory values; and (4) any kind of known or clinically proven active chronic diseases or malignancies, under previous or current disease-specific treatments.

Patients with definitive or probable vaccine-induced symptoms similar to long-COVID but without verified SARS-CoV-2 infection (i.e., lack of a positive PCR test) were excluded. Each patient had to have a negative SARS-CoV-2 PCR test before entering the outpatient medical area.

### Clinical data

Clinical and laboratory data were collected, including blood sampling, at the time of the first clinical presentation from April 2021 to May 2022, and at the second control clinical investigations in a subgroup of patients. Our pre-defined working plan for the Long COVID Outpatient care unit included (1) clinical investigations (detailed anamnesis, comorbidities, previous diseases, systemic diseases, pre-existing cardiovascular or pulmonary diseases, date of infection(s), date of vaccinations, social anamnesis); (2) blood pressure, pulse measurements and pulse oximetry; (3) previous and current medications; (4) ECG; (5) records of post-COVID investigations (e.g., chest x-ray, lung function, Holter-ECG, echocardiography, 6-min walking test, ergometry). After a comprehensive summary of the available data, the clinical diagnostics were completed with specific imaging (computed tomography, cardiac magnetic resonance imaging) or neurological investigations, if clinically indicated.

Each patient underwent extensive screening investigations to exclude objective and clinically well-defined organ diseases before the clinical diagnosis of long-COVID syndrome was made.

The clinical endotypes of long-COVID syndrome were defined in accordance with the cluster of symptoms^46,47^ and the “core outcome set” published by an international Delphi consensus^48^. Based on these references, patients were characterized as having neuropsychological, pulmonary, or cardiovascular phenotypes.

### Laboratory data

Venous blood sampling was performed at the first clinical presentation and also at a second follow-up in a subgroup of patients. The routine laboratory tests included hematologic, inflammatory, coagulation, autoimmune, endocrinologic, or tumor markers, and cardiac and other specific organ parameters (listed in Tables 1–4). Clinical virology parameters, such as virus-specific IgG or IgM, or PCR for CMV, EBV viral capsid antigen, HSV, VZV, parvovirus-B19, and EBV nuclear antigen were measured, and the results were reported qualitatively and quantitatively. All laboratory investigations were performed at the Department of Laboratory Medicine, Medical University of Vienna, Vienna, Austria. Detailed laboratory methods are described on the institution’s homepage (https://www.akhwien.at/default.aspx?pid=3985).

### Substudies

In addition to the main study comparing clinical and laboratory data between long-COVID patients who were vaccinated and non-vaccinated at presentation, we conducted three substudies, as follows: (a) to ascertain any protective effects of COVID-19 vaccination, we compared patients infected with SARS-CoV-2 after being vaccinated (“protected”) with patients who received the vaccine after SARS-CoV-2 infection (“non-protected”). (b) We analyzed data for a subgroup of patients who underwent two blood samplings (*n* = 131), comparing first and second samples overall and within subgroups, including analyzing data for a subgroup of non-vaccinated patients at presentation who later received a first vaccine after the initial blood sampling (post-presentation vaccinated, *n* = 34). (c) We compared the qualitative and quantitative DNA virus–related IgG and IgM titers between long-COVID patients and healthy unvaccinated, non-infected (pan-negative to anti-spike protein) age- and sex-matched control individuals (*n* = 105 per group).

### Statistical analyses

Continuous parameters were reported as mean ± standard deviation and nominal data as frequency with percentage (%). Several patients had quantitative IgG antibody titers over the detection limit, and the maximal detection limit value was calculated for quantitative analyses of these patients (https://bvcentre.ca/files/research_reports/08–03GuidanceDocument.pdf). Quantitative values below the detection limit (reported as lower than the detection limit) were calculated as zero. Anti-spike protein antibody was measured at the time of the first clinical presentation/blood sampling. Differences between groups were calculated using the two-sided non-parametric Mann–Whitney *U* test for data with a non-normal distribution or the two-sided Student’s *t*-test for data with a normal distribution, and the *χ*^2^ test for nominal variables.

In testing each hypothesis, we calculated statistical differences between relevant groups and analyzed only the apparent differences. Because of the exploratory nature of this study and its primary focus on hypothesis generation rather than confirmatory analysis, we employed Holm–Bonferroni corrections. For statistical analyses, SPSS Version 28.0.1.0 (142) was used. A *p* < 0.05 was considered to indicate statistical significance.

### Supplementary information

Supplementary Material

Reporting-summary

## Data Availability

The datasets used and/or analyzed during the current study are available from the corresponding author on reasonable request. All data generated or analyzed during this study are included in this published article and its supplementary information files.
