# Supplementary material for: Effect of monovalent COVID-19 vaccines on viral interference between SARS-CoV-2 and several DNA viruses in patients with long-COVID syndrome
Source: NPJ Vaccines. 2023 Sep 29;8:145. doi: 10.1038/s41541-023-00739-2 (PMC10541897; doi:10.1038/s41541-023-00739-2)

Effect of monovalent COVID-19 vaccines on viral interference between SARS-CoV-2 and several DNA viruses in patients with long-COVID syndrome

Mariann Gyöngyösi et al.

Supplementary file

Supplementary results

Effect of SARS-CoV-2 variants and vaccination schedules on clinical and laboratory data

Patient characteristics by infection with Delta versus Omicron variants were similar regarding type of vaccine applied, disease type, clinical symptoms, cumulative IgM positivity, and other laboratory parameters (Supplementary Table 1). The number of patients with Omicron infection was small (20 of the 252 patients, 7.9%), so no statistical test was performed with this group as a comparator.

Suppelemntary Table 1. Main clinical characteristics and laboratory values of the patients with Delta or Omikron variants.

	**Delta Variant**	**Omikron variant**
	**n=232**	**n=20**
Gender female	155 (66.8%)	15 (75%)
Age	44 ± 14	43 ± 14
Types of vaccine		
mRNA	115 (83.3%)	14 (82.4%)
AstraZeneca	20 (14.5%)	2 (9.1%)
Johnson&Johnson	3 (2.2%)	1 (5.9%)
Disease type		
Patient category		
1 (Neuro)	90 (38.8%)	9 (45.0%)
2 (Pulmo)	57 (24.6%)	2 (10%)
3 (Cardio)	85 (36.6%)	9 (45.0%)
Time between COVID-19 positivity and first clinical presentation (days) (median; IQR)	257 (168;378)	72 (56;98)

Any ECG Abnormalities	52 (22.4%)	9 (45%)
IgG-2 subfraction mg/dL	349 ± 140	316 ± 115
IgG-4 subfraction mg/dL	63.3 ±55.4	64.8 ±59.8
Cumulative IgM positivity	33 (14.2%)	5 (25.0%)
CMV IgG mg/dL	60.2 (0;116)	76.9 (0;116)
CMV IgM mg/dL	0 (0;0)	0 (0;5.67)
EBV IgG mg/dL	113 (336;750)	156 (57;237)
EBV IgM mg/dL	0 (0;0)	0 (0;0)
EBV EBNA IgG mg/dL	251 (68;518)	176 (39;482)
HSV IgG mg/dL	22.7 (1.1;30.0)	7.64 (0;30)
HSV IgM mg/dL	0 (0;0)	0 (0;0)
VZV IgG mg/dL	1059 (648;1567)	1448 (767;19789
VZV IgM mg/dL	0.19 (0.14;0.28)	0.18 (0.14;0.26)
Parvo-B19 IgG mg/dL	22.5 (2.58;42.8)	18 (2.4;45)
Parvo-B19 IgM mg/dL	0.23 (0;0.45)	0 (0;0)

Among the vaccinated patients, 4 of 22 (18.2%) patients who received monovalent AstraZeneca and 13 of 129 (10.1%) patients who received mRNA vaccines (1 Moderna and 12 Pfizer-BioNTech) had cumulative IgM positivity (not significant). All investigated clinical and laboratory parameters listed in Tables 1–4 were similar between the AstraZeneca and mRNA vaccination groups.

Complete versus incomplete vaccination did not show associations with clinical or laboratory data. A third vaccination (i.e., booster; n = 34) was associated with a decreased absolute value of Parvo-B19 IgM titer in serum (0.26 ± 0.28 vs 0.05 ± 0.14 mg/dL), compared with having no booster (n = 121). The number of patients receiving a booster and the number with Parvo-B19 positivity were low, so to avoid a statistical type I error, we did not perform statistical analysis of these groups.

Effect of time-sequence of vaccination first versus SARS-CoV-2 infection first

Supplemental Table 2. Clinical data for vaccinated patients with long-COVID syndrome, comparing the "non- protected" and "protected" subgroups.

Clinical data	**Patients with first infection before**	**Patients first vaccinated before**	**P value**

	**vaccination (subgroup non-protected) (n=113)**	**infection (subgroup protected) (n=42)**	
Gender female	76 (67%)	28 (67%)	
Age	44.8±14.9	41.6.9±14.7	
DM	6 (5%)	0 (0%)	
Hypertension	40 (35%)	8 (19%)	
HLP	31 (27%)	8 (19%)	
Smoking	11 (10%)	5 (12%)	
Syst RR	133±17	129±18	
RR Diast	84±9	82±16	
Heart rate (bpm)	72±11	75±13	
Patient category			
1 (Neuro)	46 (40.7%)	15 (35.7%)	
2 (Pulmo)	30 (26.5%)	8 (19.0%)	
3 (Cardio)	37 (32.7%)	19 (45.2%)	
COVID-related data			
Time between COVID-19 positivity and first clinical presentation (days) (median; IQR)	334 (258;413)	88 (53;141)	**<0.001**
Time between vaccine and first clinical presentation (days) (median; IQR)	121 (63;218)	298 (222;344)	**<0.001**
Vaccine mRNA-type	87 (71.9%)	34 (72.7%)	
Anti-spike protein titer	2500 (2500;2500)	2500 (2175;2500)	
ECG			
Any ECG Abnormalities	27 (24%)	15 (36%)	
Rhythm disturbances	5 (4%)	3 (7%)	
Conduction abnormalities	24 (21%)	14 (33%)	
QRS duration (ms)	92±15	92±11	

Differences between groups were calculated using the two-sided non-parametric Mann–Whitney U test

Supplemental Table 3. Clinical laboratory data for vaccinated patients with long-COVID syndrome, comparing the "non- protected" and "protected" subgroups.

Routine clinical lab data (normal range)	**Patients with first infection before vaccination (subgroup non-protected) (n=113)**	**Patients first vaccinated before infection (subgroup protected) (n=42)**	**P value**
**Hematology**			
Hgb g/dL	14.1±1.3	14.2±1.3	
Platelet G/L	256±56	251±56	
Leukocyte G/L	6.7±1.8	6.1±1.5	
Creatinin mg/dL	0.77±0.14	0.77±0.14	
Albumin g/L	47.3±3.0	47.9±2.7	
SGOT U/L	23±8	24±10	
SGPT U/L	26±23	30±17	
Iron ug/dL	92.0±35.1	91.7±25.5	
TSH u/U/mL	1.55±0.96	1.69±1.1	
**Coagulation**			
Prothrombin time %	99.6±19.1	99.7±19.4	
INR	1.1±0.2	1.1±0.1	
aPTT s	35.0±3.6	35.4±3.9	
Fibrinogen mg/dL	316±62	312±77	
D-dimer ug/mL	0.27 (0;0.43)	0.0 (0;0.36)	
Elevated D-dimer	19/102 (18.6%)	4/37 (10.8%)	
vWF antigen %	121±50	120±54	
ADAMTs13 activity %	104±25	112±27	
**Cardiology**			
Troponin T ng/L	0 (0;5)	0 (0;6)	
Elevated troponin T	3/109 (2.8%)	0/39 (0%)	
Creatine kinase U/L	110±71	99±45	
NT-proBNP pg/mL	50.0 (33.4;108.0)	34.9 (19.2;74.6)	**0.018**

Elevated proBNP	10/109 (9.2%)	2/40 (5.0%)	

Differences between groups were calculated using the two-sided non-parametric Mann–Whitney U test

Supplemental Table 4. Circulating inflammatory biomarkers in vaccinated patients with long-COVID syndrome, comparing the "non- protected" and "protected" subgroups

Inflammatory parameter	**Patients with first infection before vaccination (subgroup non-protected) (n=113)**	**Patients first vaccinated before infection (subgroup protected) (n=42)**	**P value**
C-reactive protein mg/dL	0.11 (0.06;0.24)	0.1 (0.04;0.20)	
LDH U/L	168 (157;188)	163 (146;185)	
Ferritin ug/L	92.9 (48.8;156.6)	70.4 (38.2;144.8)	
Transferrin mg/dL	270±46	275±41	
Transferrin saturation %	25.4±12.3	24.2±7.9	
Histamin mg/dL	7.7±3.9	6.8±2.1	
IL-6 pg/mL	1.56 (0;2.26)	1.56 (0;2.1)	
Procalcitonin ng/mL	0.03 (0;0.04)	0.03 (0;0.04)	
Total IgG mg/dL	1105±224	1079±227	
Total IgA mg/dL	203±87	186±65	
Total IgM mg/dL	108±53	103±43	
Total IgE kIU/L	31.0 (14.0;79.5)	21.4 (11.4;70.6)	
IgG-1 subfraction mg/dL	699±150	657±148	
IgG-2 subfraction mg/dL	326±110	342±136	
IgG-3 subfraction mg/dL	36.4±18.5	36.0±15.9	
IgG-4 subfraction mg/dL	53.9±45.6	52.2±45.1	

Tryptase ug/L	4.9±3.3	4.9±2.5	
Rheumafactor Latex IU/mL	0 (0;0)	0 (0;0)	
Alpha 1 antitrypsin mg/dL	137±26	129±18	
Cardiolipin IgG U/mL	1.5 (1.2;1.8)	1.5 (1.3;2.0)	
Cardiolipin IgM U/mL	1.9 (1.4;3.45)	1.5 (1.1;2.4)	**0.025**

Differences between groups were calculated using the two-sided non-parametric Mann–Whitney U test

Supplemental Table 5. Quantitative and qualitative virus parameters of vaccinated patients with long-COVID syndrome, comparing the "non- protected" and "protected" subgroups.

Routine virology lab data	Patients with first infection before vaccination (subgroup non-protected) (n=113)	Patients first vaccinated before infection (subgroup protected) (n=42)	P value
**Quantitative data**			
CMV IgG mg/dL	7.0 (0;114.5)	48.8 (0;105.6)	
CMV IgM mg/dL	0 (0;0)	0 (0;1.3)	
EBV VCA IgG mg/dL	358 (103;750)	162 (64;358)	**0.004**
EBV VCA IgM mg/dL	0 (0;0)	0 (0;0)	
EBV EBNA IgG mg/dL	247 (68;502)	121 (24;600)	
HSV IgG mg/dL	22.1 (1.8;30.0)	19.5 (0;30)	
HSV IgM mg/dL	0 (0;0)	0 (0;0)	
VZV IgG mg/dL	1061 (682;1514)	1202 (573;1684)	
VZV IgM mg/dL	0.18 (0.13;0.27)	0.18 (0.14;0.26)	
Parvo_B19 IgG mg/dL	27 (4.8;46)	23 (1.7;46)	
Parvo_B19 IgM mg/dL	0.22 (0;0.41)	0 (0;0.38)	0.066
**Qualitative data**			
Cumulative virus IgM positivity (n=155)	11 (9.7%)	6 (14.3%)	
CMV IgG positivity (n=152)	60 (54.4%)	25 (59.5%)	
CMV IgM positivity (n=152)	1 (0.9%)	3 (7.1%)	0.064
EBV IgG positivity (n=151)	109 (98.2%)	36 (90.0%)	**0.043**
EBV IgG positivity above detection limit (n=151)	40 (36.0%)	7 (17.5%)	**0.022**
EBV IgM positivity (n=151)	3 (2.7%)	0 (0%)	
EBV EBNA IgG (n=244)	99 (89.2%)	31 (77.5%)	0.063
EBV EBNA IgG above detection limit (n=151)	22 (19.8%)	10 (25.0%)	

HSV IgG positivity (n=152)	96 (86.5%)	36 (87.8%)	
HSV IgG positivity above detection limit (n=152)	38 (34.2%)	14 (34.1%)	
HSV IgM positivity (n=152)	5 (4.5%)	0 (0%)	
VZV IgG positivity (n=153)	111 (100%)	42 (100%)	
VZV IgM positivity (n=153)	2 (1.8%)	0 (0%)	
Parvo_B19 IgG positivity (n=152)	89 (80.2%)	35 (85.4%)	
Parvo_B19 IgM positivity (n=152)	4 (3.6%)	4 (9.8%)	

Differences between groups were calculated using the two-sided non-parametric Mann–Whitney U test.

Supplementary Methods

The definition of the long-COVID/post-COVID syndrome is illustrated in Supplementary Figure 1.

Limitations

Qualitative and quantitative measurements of DNA virus–related IgG and IgM were performed; if IgM was positive, a PCR test was conducted that was not always positive. Of note, IgM is positive longer than the viral load persists so that several patients had a negative PCR test despite positive IgM results. Cross-reactivity or a nonspecific immune reaction might also explain the combination of a positive IgM test coupled with a negative PCR (Ref. 37). However, our laboratory measures IgM-specific virus capsid antigen, and rheumatic disease or other immunsystem-related pathologic antibody production was excluded.

Supplementary Figure 1. Time course of COVID-19 infection, and post-acute and chronic long-COVID/post-COVID syndromes (Refs 26-29).

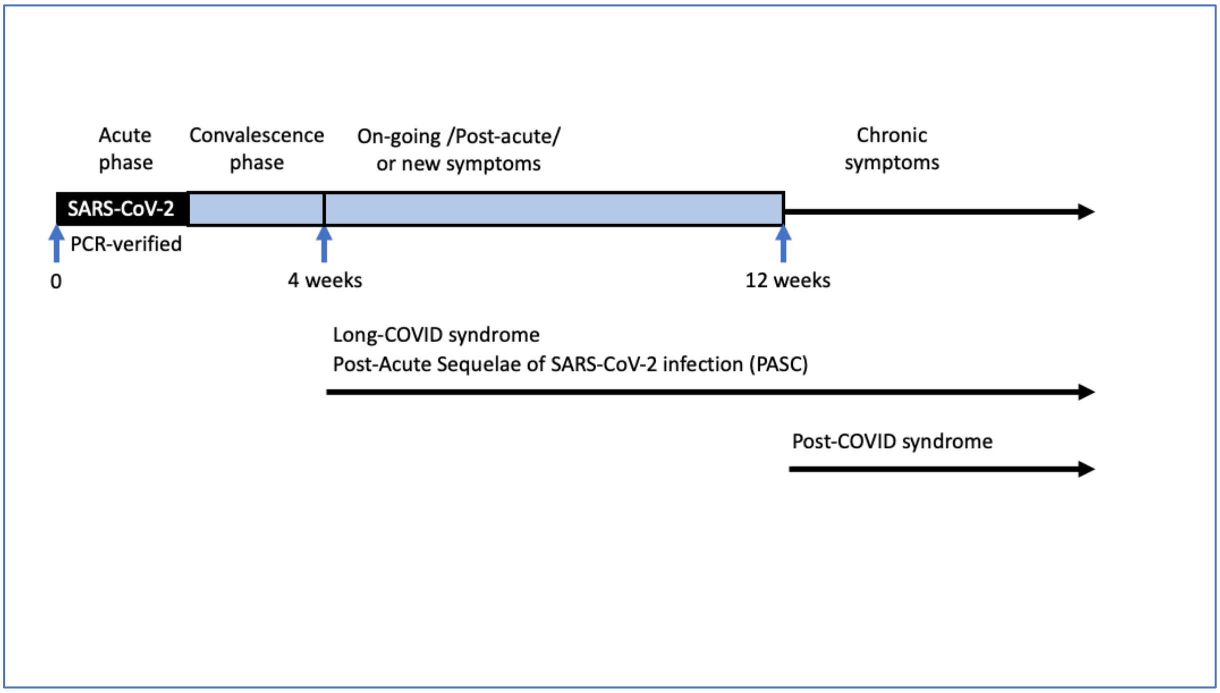

Supplementary Figure 2. Vaccination coverage of the study patients. Numbers represent the number of patients receiving the vaccines.

All patients received monovalent vaccines. Of note, the bivalent mRNA vaccine was approved by the U.S. Food and Drug Administration in April 2023, almost one year after the last patient was included in our study.

Light blue with black letters and numbers: first dose of monovalent mRNA vaccine; blue with red letters and numbers: second dose of monovalent mRNA vaccine; dark blue with white letters and numbers: third monovalent mRNA vaccine (i.e., booster). Light orange with black letters and numbers: first monovalent dose of AstraZeneca; dark orange with red letters and numbers: second dose of monovalent AstraZeneca. Green with black letters and numbers: monovalent Janssen /Johnson & Johnson vaccine. Of note, four patients who had the Astra Zeneca vaccine as their first dose received a monovalent mRNA vaccine as a second dose, and three patients had a monovalent mRNA vaccine as their third dose. Two patients were vaccinated with a "booster" mRNA after receiving the Janssen vaccine.

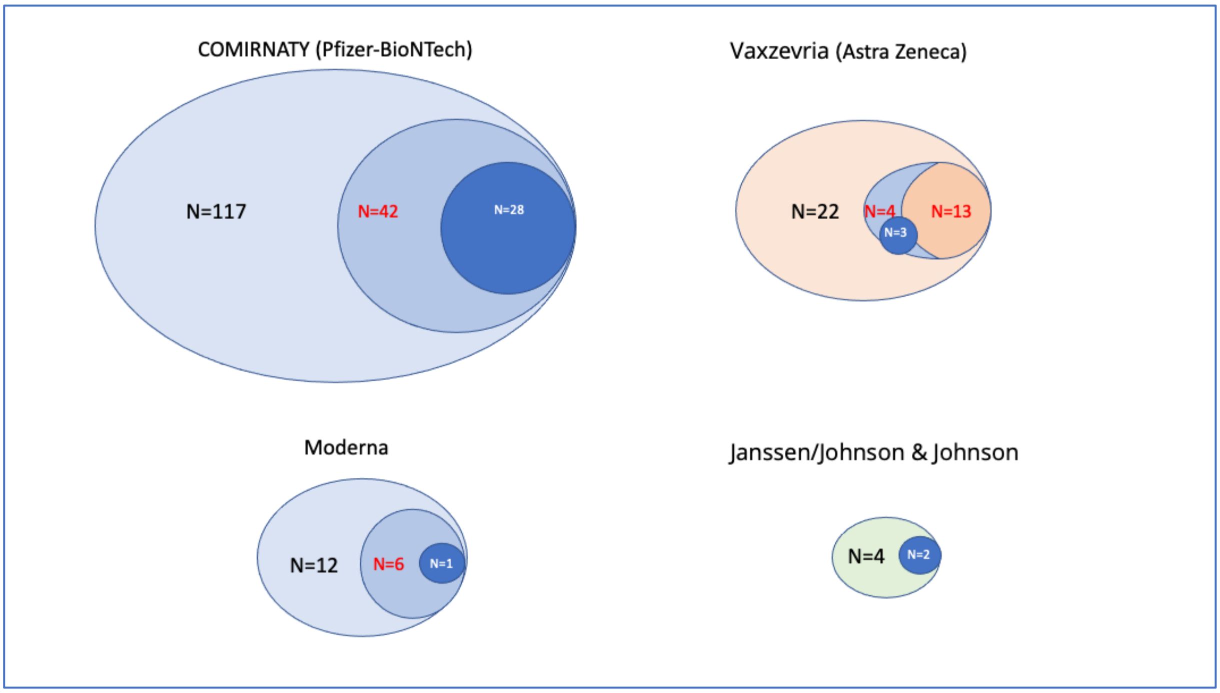

Supplement: Supplementary file 1 — Supplementary Material [file 41541_2023_739_MOESM1_ESM.pdf]
